# Transcriptome analysis in primary neural stem cells using a tag cDNA amplification method

**DOI:** 10.1186/1471-2202-6-28

**Published:** 2005-04-15

**Authors:** Maria Sievertzon, Valtteri Wirta, Alex Mercer, Konstantinos Meletis, Rikard Erlandsson, Lilian Wikström, Jonas Frisén, Joakim Lundeberg

**Affiliations:** 1 Royal Institute of Technology, AlbaNova University Center, KTH Genome Center, Department of Biotechnology, S-106 91 Stockholm, Sweden; 2 NeuroNova AB, S-114 33 Stockholm, Sweden; 3 Department of cell- and molecular biology, Medical Nobel Institute, Karolinska Institute, S-171 77 Stockholm, Sweden

## Abstract

**Background:**

Neural stem cells (NSCs) can be isolated from the adult mammalian brain and expanded in culture, in the form of cellular aggregates called neurospheres. Neurospheres provide an *in vitro *model for studying NSC behaviour and give information on the factors and mechanisms that govern their proliferation and differentiation. They are also a promising source for cell replacement therapies of the central nervous system. Neurospheres are complex structures consisting of several cell types of varying degrees of differentiation. One way of characterising neurospheres is to analyse their gene expression profiles. The value of such studies is however uncertain since they are heterogeneous structures and different populations of neurospheres may vary significantly in their gene expression.

**Results:**

To address this issue, we have used cDNA microarrays and a recently reported tag cDNA amplification method to analyse the gene expression profiles of neurospheres originating from separate isolations of the lateral ventricle wall of adult mice and passaged to varying degrees. Separate isolations as well as consecutive passages yield a high variability in gene expression while parallel cultures yield the lowest variability.

**Conclusions:**

We demonstrate a low technical amplification variability using the employed amplification strategy and conclude that neurospheres from the same isolation and passage are sufficiently similar to be used for comparative gene expression analysis.

## Background

The most frequently used method to analyse scarce RNA samples is to employ RNA amplification technology [1,2], enabling analysis of the full length transcripts. We have recently reported on an alternative transcriptome amplification method that minimises differences in transcript length in the amplification step [3,4]. This method is based on fragmentation of the mRNA (cDNA) population followed by isolation of a unique, short and representative 3'end tag of each transcript prior to amplification by PCR. Here we have evaluated and applied the methodology on neural stem cells (NSCs).

NSCs can be isolated from the fetal or adult mammalian brain and grown *in vitro *in the presence of growth factors to form floating aggregates of cells denoted neurospheres [5-7]. A neurosphere is derived from one clonally expanded NSC or progenitor cell [8]. As the original NSC or progenitor cell proliferates the new cells adhere to each other, eventually forming a neurosphere. Every neural stem cell in a neurosphere has the potential to differentiate towards a neuronal or a glial lineage depending on the internal neurosphere milieu and external signals. Neurospheres are thus complex structures consisting of many cell types that can have varying degrees of differentiation commitment, but that are all derived from the same clonally expanded cell. Neurospheres have extensive cell-cell contacts and a dense extracellular matrix. When plated onto solid support in combination with growth factor withdrawal the cells start to differentiate into all neural cell types (neurons, astrocytes and oligodendrocytes)[9]. *In vitro *expanded neural stem cells may therefore serve as an *in vitro *model of neurogenesis. The similarities between the *in vivo *and *in vitro *processes of neurogenesis are not well established although some characteristics are expected to be conserved [10] and therefore challenging a cell in *vitro *will unveil some of its developmental properties and potentials. By subjecting neurospheres to different microenvironments (e.g. through the addition or withdrawal of drugs or factors) it is possible to uncover factors and mechanisms important for proliferation or differentiation into certain cell lineages, for example neurons of a particular type [11,12]. Furthermore, NSCs expanded as neurospheres also hold the promise of becoming an important source of cells for cell replacement therapies of different neurological diseases [13,14].

Due to the great scientific interest in NSCs and the promise of their clinical use we decided to investigate NSCs from a gene expression perspective. An important aspect was to investigate if neurosphere heterogeneity [8] is reflected in their transcriptome. Neurosphere populations from different levels of technical and biological replication were analysed by taking advantage of microarrays with 5159 spotted mouse cDNA clones, in combination with a highly sensitive amplification method. We compared neurospheres cultured under identical conditions but in separate culture flasks, as well as from different passages and from parallel isolations. The results are discussed from the perspective of differences in the number and extent of differentially expressed genes.

## Results

**Figure 1:**
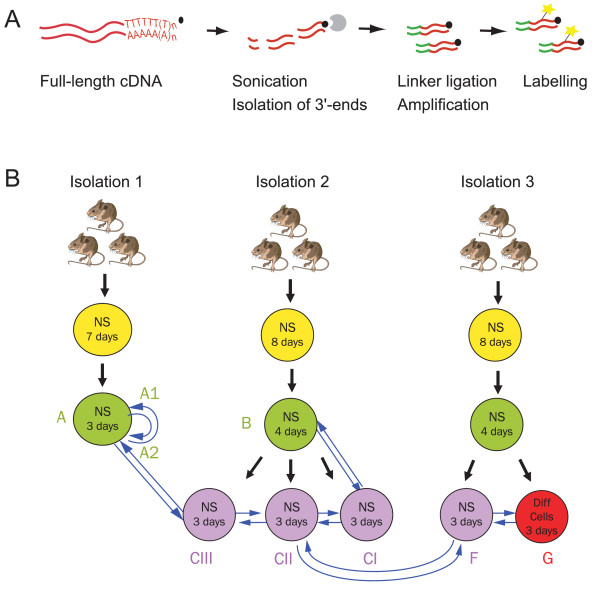
Experimental approach; A) Schematic overview of the utilised amplification protocol. For details see text. B) Experimental design. NSCs were isolated from the lateral ventricular region of brains from three pools of mice (three isolations) and grown as neurospheres. Sample G was induced to differentiate by withdrawing the growth factors from the culture medium, plating on solid support and adding serum. RNA was isolated from different passages as indicated and used for subsequent microarray hybridisations. Blue arrows represent duplicate hybridisations, arrowhead represents labelling with Cy5 and arrow tail represents labelling with Cy3.

Different sources of neurospheres were used to investigate the extent of heterogeneity between neurosphere populations at the gene expression level. To facilitate a broad transcript analysis of this relatively scarce material a recently developed amplification methodology [3,4] was used (Figure 1A) in combination with microarray technology. In brief, the approach involves biotinylation of the 3'-end of the cDNA using a biotinylated oligo(dT) primer in the first-strand cDNA synthesis reaction. The cDNA is randomly fragmented by sonication into 50–500 bp fragments. The 3'-ends (denoted 3'-end signature tags), representing the most unique part of most transcripts, are isolated by binding to streptavidin-coated beads. Linkers are ligated onto the 3'-end signature tags, which are subsequently cleaved off the beads and finally amplified using PCR. This generates a smear of random-sized fragments (data not shown) which is labelled by asymmetric PCR and then hybridised to microarrays. Here we used a mouse microarray comprising of 5159 mouse cDNA clones, printed in duplicate.

Differential expression was determined in a series of microarray experiments, as outlined in Figure 1B. Neurosphere cultures were initiated from cells dissociated from three pools of adult lateral ventricle wall tissue dissected from either 3 or 10 mice, as three identical but separate isolations. Primary neurospheres were passaged one or two times and harvested three to four days after passage. Average neurosphere size was deemed a more critical factor than the length of the incubation time, hence some neurosphere cultures were incubated for one day longer than others to obtain uniformity in neurosphere size between cultures at the key times of passaging and harvesting. When passaged twice the neurospheres were split into two or three equivalent cultures. This allowed us to measure the variability in gene expression levels between different isolations, as well as between passages and between parallel cultures. In order to estimate the technical noise, self-to-self hybridisations were performed using RNA from one of the cultures. To confirm that we were able to detect differential gene expression cells in one of the parallel cultures were induced to differentiate into neurons, astrocytes and oligodendrocytes by withdrawing the growth factors from the culture medium, plating on solid support and adding serum (in this work referred to as differentiated cells). The nomenclature of the samples is given in Figure 1B. Seven different comparisons were made; A1-A2 (technical replicate), CI-CII and CII-CIII (culture replicates), B-CI (different passages), A2-CIII and CII-FI (different isolations) and F-G (neurospheres vs. differentiated cells). The use of short-term passaged neurospheres limits the number of cells that can be generated. Consequently the amount of RNA that can be isolated is below that normally used in labelling reactions for microarray hybridisations (approximately 10 μg total RNA without amplification). After mRNA isolation and cDNA synthesis we therefore chose to amplify the obtained material using the method described above. Two replicate and two dye-swap hybridisations were performed for each comparison, adding up to four hybridisations for each comparison in total.

**Figure 2:**
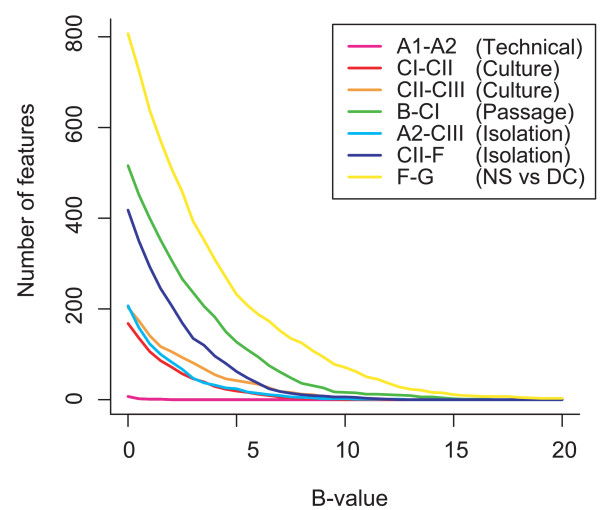
B-value distribution for each of the comparisons; The B-value is calculated through empirical Bayes statistics and scores the genes according to their probability of differential expression. Higher B-value means higher probability of differential expression. NS = neurosphere, DC = differentiated cells.

The microarray data was filtered (for details see Methods) and print-tip lowess normalised. Differentially expressed genes were identified using an empirical Bayes moderated t-test and by calculation of the associated p-values [15,16]. In the t-test the contribution of within-array replicate features was taken into account [17] and the genes were ranked according to the probability of differential expression (B-value; depending on both the fold change and the variability over the four hybridisations). Higher B-value indicates higher probability of differential expression. Genes were defined as differentially expressed if the fdr-adjusted p-value was < 0.001 (corresponding to an approximative B-value > 0.3). In Figure 2 the B-value distribution for each comparison is shown. The figure shows no differentially expressed (DE) genes in the technical replicate (using the amplification strategy), a higher number of DE genes in neurospheres cultured in parallel, an even higher number of DE genes in neurospheres from different isolations and passages, and the highest number of DE genes in neurospheres vs. differentiated cells. Note the high number of DE genes in neurospheres from the same isolation but subsequent passages (B-CI), indicating that the neurospheres may change character over time as they grow and proliferate *in vitro*.

**Figure 3:**
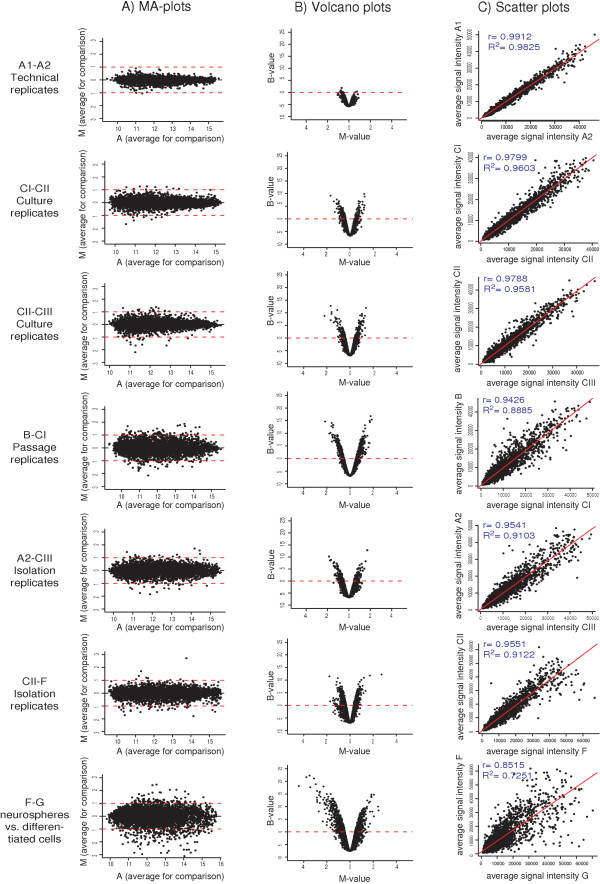
Graphs displaying the variability of the data at different levels of replication; In all graphs one dot represents one gene. Panel A) shows MA-plots for each comparison. The x-axis represents the intensity of the feature (A = 1/2log_2_(Cy5*Cy3)). The y-axis represents the magnitude of differential expression of the gene (M = log_2_(Cy5/Cy3)), calculated after filtration and normalisation of the data. Dotted lines are drawn at M-values 1 and -1, i.e. at a 2-fold difference in signal intensity between the compared samples. Panel B) shows volcano plots for each sample. The x-axis shows the M-value for each gene and the y-axis the corresponding B-value (calculated by empirical Bayes moderated t-test) for that gene. Panel C) shows scatter plots for each comparison. The x-axis displays the average signal intensity for one sample and the y-axis the average signal intensity for the other sample. Also shown are the values of the Pearson correlation coefficient (r) and the coefficient of determination (R^2^).

To further investigate the variability in gene expression between the different neurosphere samples we visualised the data using a series of plots displayed in Figure 3. In panel A) the average A-value (1/2log_2_(sample X intensity * sample Y intensity)) for each gene is plotted against the corresponding M-value (log_2_(sample X intensity / sample Y intensity)). The plots show that after filtration and normalisation there is no intensity bias in the distribution of DE genes. They also show that the M-values for the technical replicates (A1-A2) are collected and centred close to zero (corresponding to a ratio of 1), whereas the spreading of the M-values are higher for culture replicates (CI-CII and CII-CIII), passage replicates (B-CI) and isolation replicates (A2-CIII and CII-F). The highest spreading of M-values can be seen for the neurosphere vs. differentiated cells hybridisations (F-G), where many genes have M-values between +/-1 and +/-3 (corresponding to a fold change of 2 to 8). In panel B) the B-value for each gene is plotted against the corresponding M-value. By definition genes with a high M-value will obtain a higher B-value, which gives the plots the characteristic volcano shape. Also here it is clear that the technical replicates have no statistically significant DE genes (with high B-values), whereas culture, passage and isolation replicates have several genes with high B-values, and the neurosphere vs. differentiated cells comparison clearly has the highest number of DE genes. This is further visualised in Figure 3, panel C), where the average of the signal intensity for each gene and sample is plotted against the average signal intensity for that gene in the other sample. Once again the Pearson correlation for the two samples is highest for the technical replicates (r = 0.99), lower for the culture replicates (r = 0.98 and r = 0.98 respectively), passage replicate (r = 0.94) and isolation replicates (r = 0.95 and r = 0.96 respectively) and lowest for the neurosphere vs. differentiated cells (r = 0.85).

**Figure 4:**
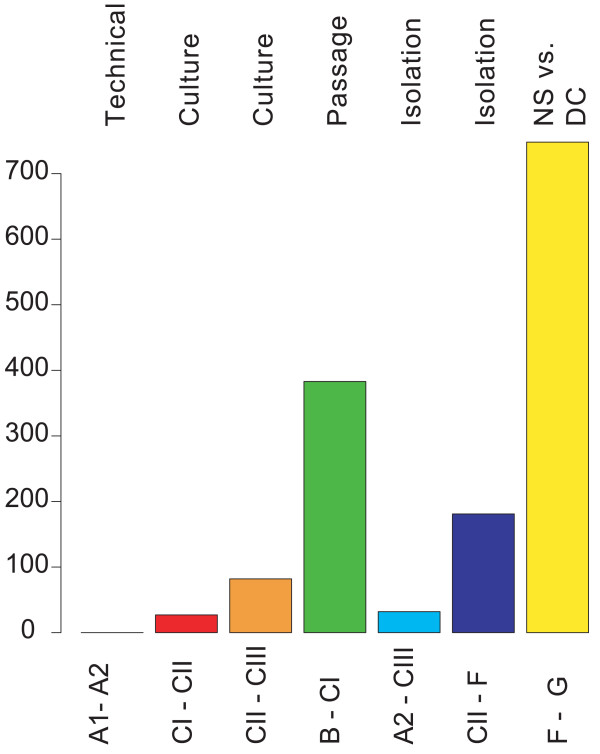
The number of differentially expressed genes in each comparison; Genes with p < 0.001, calculated by empirical Bayes moderated t-test and false discovery rate adjustment, are included. NS = neurosphere, DC = differentiated cells.

The number of differentially expressed genes in each comparison is summarised in Figure 4. Genes with a false discovery rate (fdr) adjusted p-value < 0.001, giving less than one false positive per 1000 genes, are included. This further demonstrates that the lowest number of DE genes is in the technical replicates and the highest number in the comparison of the neurospheres vs. differentiated cells (748 genes). Again, the most noteworthy result is that neurospheres of different passages (B-CI) have a surprisingly high number of DE genes (383 genes) compared to the other comparisons. To further explore the magnitude of differential expression of these genes (with p < 0.001) a table of their distribution over fold change was made (Table 1). As expected, a high number of genes have high fold changes for the passage and isolation replicates and the neurosphere vs. differentiated cells comparison, whereas fewer genes are within the higher fold change ranges for the culture replicates.

**Table 1:** Distribution of differentially expressed genes over fold change.

M interval	Fold change	A1-A2	CI-CII	CII-CIII	B-CI	A2-CIII	CII-F	F-G
+/-(0–0.5)	0–1.4						17	16
+/-(0.5–1)	1.4–2		14	51	260	7	147	455
+/-(1–1.5)	2–2.8		12	26	108	20	14	190
+/-(1.5–2)	2.8–4		1	4	14	5	2	52
+/-(2–2.5)	4–5.6			1	1			19
+/-(>2.5)	>5.6						1	16

Total p < 0.001 (fdr)		0	27	82	383	32	181	748

Genes with p < 0.001, calculated by empirical Bayes moderated t-test and false discovery rate adjustment, are included. (M = log_2_(Cy5/Cy3)).

**Figure 5:**
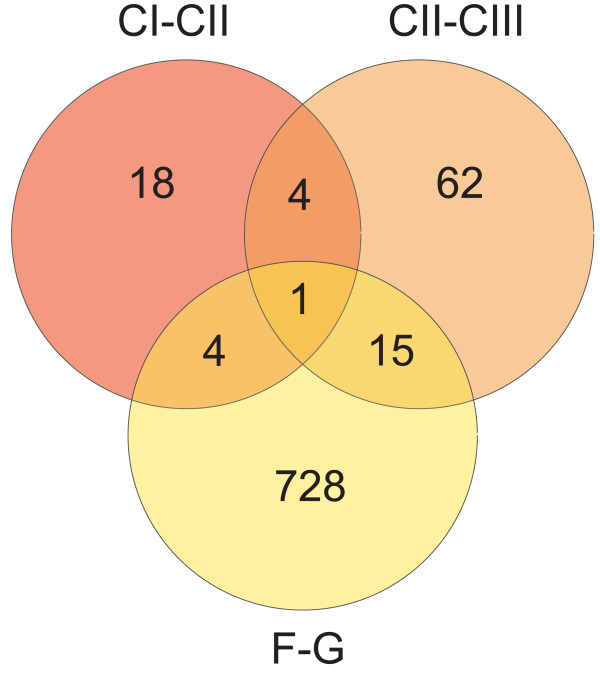
Overlap of differentially expressed genes; Compared are the results from the parallel cultures (CI-CII and CII-CIII) and the neurosphere vs. differentiated cells comparison (F-G). Genes with p < 0.001, calculated by empirical Bayes moderated t-test and false discovery rate adjustment, are included.

To investigate whether the transcript level differences in the two culture-to-culture comparisons are consistent or random events a Venn diagram was created in Figure 5, displaying the number of shared and unique DE genes in the CI-CII, CII-CIII and F-G comparisons. Five genes out of 27 (CI-CII) and 82 (CII-CIII) overlap, equivalent to 19% and 6% respectively. The vast majority of DE genes are thus not shared between the two comparisons, indicating non-systematic changes in gene expression. When compared to the neurosphere vs. differentiated cells gene list only one of the five genes is in common, further demonstrating random differences between cultures.

A list of the DE genes found in the neurosphere vs. differentiated cells comparison (F-G) is provided as an additional data file 1: Differentially expressed genes in neurosphere vs. differentiated cells comparison (the complete results for all comparisons are available in ArrayExpress using experiment accession number E-MEXP-297). The genes that show four-fold or greater fold change (M ≥ |2|) and adjusted p-values < 0.001 are shown in Table 2. This demonstrates that genes found in the F-G comparison are genes that are expected to be involved in the differentiation of neurospheres. For example, included are several myelin related genes such as *myelin-associated oligodendrocytic basic protein *(Mobp), *myelin basic protein *(Mbp) and *myelin-associated glycoprotein *(Mag), all of which are up-regulated in the differentiated sample. Also, there are some genes related to transmitter substances and their signaling; *gamma-aminobutyric acid (GABA-A) receptor*, *subunit beta 1 *(Gabrb1)and *guanine nucleotide binding protein*, *alpha o *(Gnao1), which is involved in dopamine signaling [18]. *Distal-less homeobox 1 *(Dlx1), is also widely expressed in the brain and is involved in brain development and neural differentiation [19-21].

**Table 2:** [Supplementary-material S1]Genes differentially expressed in the neurosphere vs. differentiated cells comparison (F-G).

Genbank Acc.no.	Unigene ClusterID	GeneID	Gene Name	M	B
CX240827	Mm.200608	12759	Clusterin	-3,503	22,292
CX241145	Mm.289645	17263	GTL2, imprinted maternally expressed untranslated mRNA	-3,774	21,684
CX238761	Mm.354720	14681	Guanine nucleotide binding protein, alpha o	-3,112	21,128
CX241318	Mm.289645	17263	GTL2, imprinted maternally expressed untranslated mRNA	-4,429	18,116
CX235874	Mm.40461	17433	Myelin-associated oligodendrocytic basic protein	-2,961	18,854
CX236810	Mm.210815	20411	Sorbin and SH3 domain containing 1	-2,287	17,999
CX241478	Mm.25874	77976	RIKEN cDNA B230104P22 gene	-3,435	17,660
CX241468	Mm.4543	13390	Distal-less homeobox 1	-2,843	16,192
CX240901	Mm.271178	67166	ADP-ribosylation factor-like 10C	-2,606	15,924
CX238347	Mm.252063	17196	Myelin basic protein	-2,622	15,450
CX240173	Mm.291442	20692	Secreted acidic cysteine rich glycoprotein	-2,008	14,973
CX240709	Mm.30035	107747	Formyltetrahydrofolate dehydrogenase	2,065	14,714
CX238313	Mm.206505	21858	Tissue inhibitor of metalloproteinase 2	-2,669	14,533
CX236462	Mm.34126	74617	Serine carboxypeptidase 1	-2,544	13,912
CX242347	Mm.271770	433496	Similar to Myl9 protein	-2,860	13,549
CX243886	Mm.289645	17263	GTL2, imprinted maternally expressed untranslated mRNA	-2,213	13,547
CX235484	Mm.178246	216848	Chromodomain helicase DNA binding protein 3	-2,313	12,737
CX241157	Mm.194225	70397	RIKEN cDNA 1110020A09 gene	-2,045	12,600
CX243757	Mm.260601	242521	Kelch-like 9 (Drosophila)	-2,848	12,607
CX237579	Mm.40461	17433	Myelin-associated oligodendrocytic basic protein	-2,000	11,957
CX242456	Mm.291442	20692	Secreted acidic cysteine rich glycoprotein	-2,835	11,842
CX238326	Mm.1426	51791	Regulator of G-protein signaling 14	-2,193	11,543
CX239776	Mm.252063	17196	Myelin basic protein	-2,606	11,414
CX235557	Mm.241355	17136	Myelin-associated glycoprotein	-2,220	11,257
CX239390	Mm.213204	80906	Kv channel-interacting protein 2	-2,126	10,915
CX239520	Mm.21549	80888	Heat shock 27 kDa protein 8	-2,104	10,657
CX238589	Mm.226704	14400	gamma-aminobutyric acid (GABA-A) receptor, subunit beta 1	-2,039	10,744
CX242889	Mm.29358	67971	RIKEN cDNA 2700055K07 gene	-2,032	10,827
CX235601	Mm.4857	12323	Calcium/calmodulin-dependent protein kinase II, beta	2,418	10,025
CX242440	Mm.121920	20660	Sortilin-related receptor, LDLR class A repeats-containing	-2,180	8,923
CX240196	Mm.181959	13653	Early growth response 1	2,469	8,456
CX242602			#N/A	-2,692	8,087
CX240394	Mm.18830	77569	RIKEN cDNA 3732412D22 gene	-2,078	7,216
CX237453	Mm.181959	13653	Early growth response 1	2,059	6,141
CX244387	Mm.211275	76441	Dishevelled associated activator of morphogenesis 2	-2,347	4,185
CX236987	Mm.329668	20743	Spectrin beta 3	-2,014	2,723

Genes with p < 0.001, calculated by empirical Bayes moderated t-test and false discovery rate adjustment, and fold change ≥ 4 (-2 ≥ M ≥ 2), are included. Genes with M-value < 0 are up-regulated in differentiated cells, genes with M-value > 0 are up-regulated in neurospheres. M = log_2_(Cy5/Cy3), B = B-value calculated by empirical Bayes moderated t-test. Higher B-values mean higher probability for differential expression. For a full list of differentially expressed genes in the F-G comparison see additional data file 1: Differentially expressed genes in neurosphere vs. differentiated cells comparison.

To understand the biological significance of the overall changes in gene expression, DE genes in the neurosphere vs. differentiated cells comparison (F-G) were categorised according to their gene ontology theme annotation. Genes with an adjusted p < 0.001 were included in the analysis and the probability of a theme being over-represented in the data was calculated using Jackknife Fisher's exact probability test implemented in the EASE software [22]. Table 3 shows the most highly represented gene ontology themes (of category biological function). Themes that showed enrichment in differentiated cells include neurogenesis, synaptic transmission, cell-cell signalling and development. In contrast, themes that showed enrichment in neurospheres include electron transport and mitotic cell cycle.

**Table 3:** The most highly represented gene ontology themes in the neurosphere vs. differentiated cells comparison (F-G).

Biological process	no of genes on array	no of DE genes in F-G	enriched in NS	enriched in DC	Fisher Exact
cell adhesion	56	17	6 (35%)	11 (65%)	0.00939
lipid metabolism	71	20	8 (40%)	12 (60%)	0.0123
neurogenesis	35	12	2 (17%)	10 (83%)	0.0101
mitotic cell cycle	30	10	7 (70%)	3 (30%)	0.0225
synaptic transmission	35	11	2 (18%)	9 (82%)	0.0266
development	166	37	8 (22%)	29 (78%)	0.0429
neuromuscular physiol. process	37	11	2 (18%)	9 (82%)	0.0396
transmission of nerve impulse	37	11	2 (18%)	9 (82%)	0.0396
electron transport	38	11	8 (73%)	3 (27%)	0.0476
endocytosis	24	8	2 (25%)	6 (75%)	0.0399
organismal movement	43	12	3 (25%)	9 (75%)	0.0511
siderochrome transport	34	10	4 (40%)	6 (60%)	0.0522
alcohol metabolism	39	11	4 (36%)	7 (64%)	0.0565
neurophysiological process	49	13	3 (23%)	10 (77%)	0.0626
organismal physiological process	86	20	5 (25%)	15 (75%)	0.0842
energy pathways	36	10	7 (70%)	3 (30%)	0.0739
cell-cell signaling	41	11	2 (18%)	9 (82%)	0.0777
Organogenesis	89	20	4 (20%)	16 (80%)	0.112

Analysis is restricted to themes of category biological function. Genes with p < 0.001, calculated by empirical Bayes moderated t-test and false discovery rate adjustment were included in the analysis. Shown is the total number of genes found in the respective theme, as well as the number of those genes that are enriched in neurospheres (NS) and differentiated cells (DC) respectively. Jackknife Fisher's exact probability test was used to identify over-represented themes in the data. Corresponding p-values are listed.

## Discussion

This study has taken advantage of a recent template amplification method to study neurospheres at the level of transcription. RNA from different isolations, cultures and passages was isolated, amplified and analysed by microarrays. The comparison was performed by analysis of the number of differentially expressed genes for the different conditions. The results show excellent performance of the amplification protocol. No differentially expressed genes were found in the technical replicates indicating that methodological noise in all comparisons should be considered minor.

### Fluctuations of transcript levels in different populations of neurospheres

The array results for the different neurosphere conditions were much more divergent than the technical replications and we observe a varying degree of heterogeneity among the different neurosphere populations, obtained from different isolations of adult mouse lateral ventricle wall tissue, from different passages and from parallel cultures. The results show that there is a large variation in gene expression between neurospheres from different isolations as well as between neurospheres from the same isolation but from different passages. Neurospheres have previously been shown to gain altered properties through extensive, long-term passaging (more than 10 passages) [23]. Short-term passaged neurospheres have been considered rather stable, with unaltered multipotency and capacity for self-renewal [24]. Here we have shown that already between passages one and two neurospheres show altered gene expression with up to 383 DE genes (p < 0.001). Whether this is due to different properties of the parental, clonally expanded cell(s) giving rise to the neurospheres in each passage or some other reason needs to be further investigated.

Parallel culturing of neurospheres from the same isolation and the same number of passages, grown in identical conditions, show fewer DE genes (up to 82 genes, p < 0.001) than neurospheres compared between passages (383 genes, p < 0.001). Furthermore, when neurospheres are induced to differentiate and compared to undifferentiated neurospheres cultured in parallel, the number of genes DE as well as the magnitude of the M-values are clearly higher (748 genes, p < 0.001). These data indicate that an extended 3–4 day culturing, *per se*, is sufficient to induce changes in gene expression, but with careful experimental design and an appropriate number of biological replicates neurospheres cultured in parallel, from the same isolation and passage, may be used to study for example the effect of exposure to different microenvironments on gene expression.

The gene expression heterogeneity of neurospheres may be related to a number of different factors such as the age of the animal from which they were isolated, neurosphere size and the identity of the first clonally expanded cell [25]. Our results are also confirmed by observations by Suslov and co-workers that examined the expression of 16 transcripts from single neurospheres of different sizes [8]. The obtained information was used to cluster the individual neurospheres according to similar gene expression pattern. It revealed an inter-clonal heterogeneity that might reflect the maturity and developmental commitment of the parental clonogenic cell, as well as the size of the neurosphere and its time in culture. In another study it was shown that populations of neurospheres from different regions of the brain as well as from different species differ in properties such as growth rate, neuronal production and cell morphology [26].

### Genes expressed in neurospheres

The different neurosphere populations show heterogeneity in their expression profiles, yet many of the genes expressed are representative of a neurosphere transcript signature. As described earlier, neurospheres consist of several cell types of varying degrees of differentiation, a dense extracellular matrix and extensive cell-cell contacts. Electron-microscopy studies of rat fetal striatum EGF-expanded neurospheres [27], have shown that they consist of two types of cells, electron-dense and electron-lucent cells, both of which could be either healthy, apoptotic or necrotic. These neurosphere cells also demonstrated an expression of the cell adhesion molecules *E*- and *N-cadherin *(Cdh1 and Cdh2), *α- and β-catenin *(Catna1, LOC297357 and RGD:70487) and growth factor receptors for *epidermal growth factor *(Egfr) and *fibroblast growth factor *(Fgfr1), as well as *fibroblast growth factor 2 *(Fgf2). Also neurospheres from adult human brain have been characterised, revealing the same type of heterogeneous, complex structure [28,7]. These express a variety of different markers, such as *nestin *(NES; neural stem/progenitor and immature glial marker), *vimentin *(VIM; immature glia), *glial fibrillary acidic protein *(GFAP; astrocytes), *β-III-tubulin *(TUBB3; neuronal marker) and *cell adhesion molecule L1 *(L1CAM; neuronal marker), *proteolipid protein 1 *(PLP1; oligodendrocytes), *B-cell CLL/lymphoma 2 *(BCL2; anti-apoptotic), *paired box gene 6 *(PAX6; a developmentally regulated gene) and *tenascin C *(TNC; extracellular matrix protein). In our study the genes related to these phenotypes and markers are expressed at similar levels in all neurosphere replicates (CI-CII, CII-CIII, B-CI, A2-CIII and CII-F). For example we observe many genes involved in apoptosis; *Bcl2-associated X protein *(Bax), *Bcl2-associated athanogene 1 *(Bag1), *cytochrome c-1 *(Cyc1), *death associated protein 3 *(Dap3), *programmed cell death 6 interacting protein *(Pdcd6ip) and *phosphoprotein enriched in astrocytes 15 *(Pea15). Expressed are also *α-E-catenin *(Catna1), *β-catenin *(Catnb) and *fibroblast growth factor 3 *(Fgfr3), and other neurosphere markers such as *nestin *(Nes), *glial fibrillary acidic protein *(Gfap), *β-III-tubulin *(Tubb3) and *proteolipid protein 1 *(Plp1) (The complete data set is available in ArrayExpress using experiment accession number E-MEXP-297).

The list of DE genes in the neurosphere vs. differentiated cells comparison (Table 2 and additional data file 1: Differentially expressed genes in neurosphere vs. differentiated cells comparison) as well as an overview of the corresponding gene ontology classification (Table 3) also demonstrates the anticipated differences between neurospheres and differentiated neurospheres.

### Summary

The genes observed to be differentially expressed in identical but parallel cultures appear to be random, shown by the low overlap in DE genes between the two parallel culture comparisons (Figure 5). The number of erroneously identified DE genes, due to biological fluctuations, could hence be lowered by increasing the number of biological replicates. Hereby random differences will be removed and true DE genes can be selected by statistical means. It should be noted that the random differences mainly correspond to small fold changes as compared to the larger changes in the neurospheres vs. differentiated cells. Reliable differences in gene expression could therefore be obtained and studied without increasing the number of replicates if a higher cut-off for DE genes, such as fold change > 2, was chosen.

## Conclusions

We have shown that the tag cDNA amplification method is well suited for the analysis of neurospheres, demonstrating low technical variability. Furthermore we have demonstrated large differences between passages of neurospheres, but less variability between parallel cultures. The described variability appears to be random and the underlying cause(s) needs further investigations. The neurosphere variability can be addressed by increasing the number of biological replicates and careful experimental design, which will facilitate future use of neurospheres as a tool to study gene expression changes involved in neurogenesis.

## Methods

### Adult mouse neural stem cell culture

Three adult mouse neural stem cell cultures were initiated, the first originating from tissue isolated from ten mice (Culture 1) while the second (Culture 2) and third (Culture 3) cultures originated from three mice each. For each culture, identical dissection, dissociation and culture protocols were used. Briefly, the lateral wall of the lateral ventricle of 5–6-week-old mice was enzymatically dissociated in 0.8 mg/ml hyaluronidase and 0.5 mg/ml trypsin in Dulbecco's modified Eagle medium (DMEM) containing 4.5 mg/ml glucose and 80 U/ml DNase at 37°C for 20 min. The cells were gently triturated and mixed with three volumes of neurosphere medium (DMEM/F12, B27 supplement, 12.5 mM HEPES pH7.4) containing 20 ng/ml EGF, 100 U/ml penicillin and 100 μg/ml streptomycin. After passing through a 70-μm strainer, the cells were pelleted at 160 × g for 5 min. The supernatant was subsequently removed and the cells resuspended in neurosphere medium supplemented as above, plated in uncoated culture dishes and incubated at 37°C. Neurospheres were ready to be split 7–8 days after plating.

To split neurosphere cultures, neurospheres were collected by centrifugation at 160 × g for 5 min. The neurospheres were resuspended in 0.5 ml Trypsin/EDTA in HBSS (1x), incubated at 37°C for 2 min and triturated gently to aid dissociation. Following a further three-min incubation at 37°C and trituration, 3 volumes of ice-cold neurosphere medium containing EGF were added. The cells were pelleted at 220 × g for 4 min, resuspended in fresh neurosphere medium supplemented with 20 ng/ml EGF.

From Cultures 1, 2 & 3, dissociated cells were plated and grown in neurosphere medium supplemented with EGF for a further 3–4 days by which time secondary neurospheres had developed. The secondary neuropheres originating from Culture 1 were harvested for mRNA isolation (Sample A). Approximately a quarter of the secondary neurospheres originating from Culture 2 were also taken for mRNA isolation (Sample B), while the remainder were dissociated and replated in three equal fractions (100,000 cells / well (6 well plate)), cultured in neurosphere medium supplemented with EGF for 3 days, and harvested for mRNA isolation (Samples CI, CII, CIII). Secondary neurospheres originating from Culture 3 were dissociated and divided into two fractions. The first fraction was replated (100,000 cells / well (6 well plate)) and cultured identically to the cells generating Samples CI, CII & CIII. After 3 days, the cells were harvested for mRNA isolation (Sample F). The second fraction was replated in neurosphere medium supplemented with 1% fetal calf serum (FCS) onto poly-D-lysine plates to which the cells adhered. After incubating, overnight FCS concentration was reduced to 0.5%, and the cells cultured a further 2 days before centrifugation and subsequent mRNA isolation (Sample G, differentiated cells). All experiments were approved by the Karolinska Institute Ethical Committee.

### cDNA synthesis

Messenger RNA was isolated using Dynabeads^® ^mRNA DIRECT™ Kit from Dynal (Dynal A.S., Norway), according to the manufacturer's instructions. First- and RNaseH dependent second-strand cDNA synthesis (SuperScript Choice System for cDNA Synthesis) was performed according to the manufacturer's instructions (Invitrogen, CA, USA) using 45 pmol biotinylated *Not*I-oligo(dT) primer (5'-biotin-GAGGTGCCAACCGCGGCCGC (T)_15_-3'). The cDNA was phenol-chloroform extracted and ethanol precipitated and the pellet was dissolved in 40 μl of 1 × TE (10 mM Tris-HCl, 1 mM EDTA). Excess *Not*I-oligo(dT) primer was removed by Chromaspinn TE-100 column (Clontech, CA, USA).

### Amplification of 3'-end signature tags

The cDNA was fragmented and amplified according to a protocol previously described [3,4]. Shortly, fragmentation of the cDNA was performed in 40 μl 1 × TE using an inverted sonication probe, using 16 × 10 sec pulses at 90% effect (Sonifier^® ^B-12, Branson Sonic Power Company, CT, USA). Biotinylated 3'-end signature tags from the fragmented cDNA population were isolated onto 20 μl of paramagnetic streptavidin-coated beads (10 mg/ml) (Dynal A.S.) in 40 μl sample plus 40 μl Binding/Washing buffer (2 M NaCl, 0.1% Tween 20 in 1 × TE, pH 7.7) at 37°C for one hour with rotation. The immobilised signature tags were end repaired using 1.5 U T4 DNA polymerase (New England BioLabs, MA, USA) in a 30-μl reaction volume at 12°C for 20 minutes according to the supplier's recommendations. Blunt-end adapters (Sima18: 5'-GGATCCGCGGTG-3'; Sima19: 5'-TCTCCAGCCTCTCACCGCGGATCC-3') were pre-annealed and ligated onto the immobilised repaired 3'-end signature tags using a solution comprising 1.1 nmol adapter, ligase buffer (66 mM Tris-HCl, pH 7.6, 5 mM MgCl_2_, 5 mM DTT, 50 μg/ml BSA), 0.2 mM ATP, 1200 U T4 DNA ligase (New England BioLabs) in a final volume of 60 μl. Ligation was performed overnight at room temperature with constant rotation to keep beads in suspension. The signature tags were released from the magnetic beads by restriction with *Not*I (New England BioLabs) for 2 hours in a volume of 60 μl while keeping the beads in suspension. Five micro litres of the eluate containing the 3'-end signature tags was used as template in a subsequent PCR. The PCR was performed in 100 μl containing 200 μM of each dNTP, 0.75 μM Sima19, 0.75 μM *Not*I-oligo(dT) primer, 65 mM Tris-HCl pH 8.8, 4 mM MgCl_2_, 16 mM (NH_4_)_2_SO_4_, 0.5 μM BSA and 3 U AmpliTaq DNA polymerase (Perkin Elmer, MA, USA). Cycling was performed according to the following procedure, initial incubation at 72°C for 3 min, followed by addition of *Taq *DNA polymerase and subsequent cycling: 72°C for 20 min, 95°C for 1 min, 45°C for 5 min, 72°C for 15 min, followed by four cycles (95°C for 1 min, 50°C for 1 min, 72°C for 15 min), and 13 cycles (as previously optimised) (95°C for 1 min, 50°C for 1 min, 72°C for 2 min).

### Target labelling and microarray hybridisation

The 3'-end signature tags were purified using QIAquick^® ^PCR purification kit (Qiagen, Germany). Direct labelling was performed using Cy3-dCTP or Cy5-dCTP (Perkin Elmer, MA, USA) in a linear, asymmetric PCR. The reaction was performed in a 50-μl labelling mix containing 100–200 ng purified 3'-end signature tags, 80 μM dATP, dGTP and dTTP, 20 μM dCTP, 5 μM Sima19 primer, 2 mM MgCl_2_, 1 × PCR Buffer II (Applied Biosystems, Ca, USA), 3 U AmpliTaq Gold^® ^(Applied Biosystems) and 60 pM Cy3-dCTP or Cy5-dCTP. The labelling mix was cycled as follows: 95°C for 12 min, then 20 cycles (95°C for 30 sec, 50°C for 30 sec, 72°C for 10 min). Excess primer and nucleotides were removed using QIAquick^® ^PCR purification kit (Qiagen). The eluted labelling products were speed vacuumed until dry, then dissolved in 55 μl hybridisation buffer (24% formamide, 5 × SSC and 0.1% SDS) (20 × SSC contains 3 M NaCl and 0.3 M Na_3_citrate × 2H_2_O). Cy3 and Cy5 labellings were blended and mixed with 25 μg human Cot-1 DNA (Invitrogen) and 50 μg polyA DNA (Operon Biotechnologies GmbH, Germany). The arrays (ArrayExpress accession number E-MEXP-297, submission in progress) [29] contained 5169 probes originating from a lateral ventricle wall cDNA library (clone library "Mus Musculus Lateral Ventricle Wall C57BL/6 adult") and a set of control features all printed in duplicate. Details regaring the array manufacturing are available through ArrayExpress. Briefly, probes were generated through PCR amplification and subsequently purified using Multiscreen-384 filter plates (Millipore). Purified products in 50% DMSO were printed onto GAPS-II slides (Corning Inc) using the QArray arrayer (Genetix) and attached using 250 mJ UV-light (Stratalinker). The arrays were first prehybridised for 30 min in a 42°C prehybridisation solution (1% BSA, 5 × SSC, 0.1% SDS), then washed in water and isopropanol and dried through centrifugation. The sample was denatured in 95°C for 3 min, then applied to the array and incubated in a hybridisation chamber in 42°C for 18 hours. After hybridisation the arrays were washed in three successive wash buffers with increasing stringency: (1) 1 × SSC and 0.2% SDS, 42°C, (2) 0.1 × SSC and 0.2% SDS, room temperature, (3) 0.1 × SSC, room temperature. All wash steps were made on a shaking table for 4 min. After the last step the array was immediately centrifuged in a slide centrifuge and kept in the dark until scanning. Scanning was performed using the GMS 418 Array Scanner from Genetic MicroSystems (Affymetrix Inc, CA, USA).

### Image and data analysis

All image and data analysis steps were conducted in GenePix Pro 4.1.1.4 (Axon Instruments Inc, CA, USA) or R [30]. The analysis in R was carried out using Bioconductor [31], LIMMA [16], aroma [32] and the kth-package [33]. The analysis was conducted according to the following workflow. (1) Image tiff-files were created by scanning the microarrays with the GMS 418 Array Scanner. (2) Feature identity and foreground/background intensities were extracted from the tiff files using GenePix Pro 4.1.1.4. (3) GenePix result files were imported into R and gene expression measurements were obtained for each feature by subtracting the median of the local background from the median of the foreground signal. (4) A filter was used to identify and correct for features that had one channel (Cy3 or Cy5) below the background or at zero and one channel stronger than the background. The signal in the weaker channel was for these spots set to one plus the intensity of the local background. Features with both channels below the background or at zero were removed from the data set. (5) A second filter was used to remove features that were saturated in both channels. (6) A third filter was used to remove features with abnormal size (below 110 and above 230 μm in diameter). (7) A fourth filter was used to remove features where both signals had more than 70% of the pixels in the feature below the local background signal plus two standard deviations. (8) The last filter was used to remove features that were flagged as not found by GenePix. (9) Filtered data was normalised separately for each individual block on the slide using a robust local regression, print-tip lowess normalisation [34]. (10) An empirical Bayes moderated t-test [15-17] was used to rank the genes according to evidence of differential expression. The obtained p-values were adjusted for multiple testing using the false discovery rate adjustment [35] implemented in R. A p-value of less than 0.001 was considered significant and the associated gene termed differentially expressed (DE). The experimental design included reciprocal dye label assignments. These were swapped prior to the moderated t-test so that in each comparison the genes in the sample with an abbreviation that comes earlier in alphabetical order (e.g. B in B vs. CI) have positive M-values if they have a higher expression level.

## List of abbreviations

NSC; neural stem cell

DE; differentially expressed

NS; neurosphere

DC; differentiated cells

fdr; false discovery rate

## Authors' contributions

MS participated in the design of the study, drafted the manuscript, coordinated and carried out microarray experiments as well as performed data processing and data analysis. VW coordinated and carried out the manufacturing of the microarrays, performed data analysis and statistical analysis as well as assisted with the manuscript. AM participated in the design of the study, cultured cells and assisted with the manuscript. KM dissected the lateral ventricle wall tissue, isolated mRNA and coordinated the cDNA library construction for the array manufacturing, as well as assisted in writing of the manuscript. RE participated in the clone selection for the microarrays. LW conceived of the study and participated in its design and coordination. JF conceived of the study, participated in its design and coordination and assisted with the manuscript. JL conceived of the study, participated in its design and coordination of the study and helped to draft the manuscript, principle investigator. All authors read and approved the final manuscript.

## Supplementary Material

Additional File 1 Genes differentially expressed in the neurosphere vs. differentiated cells comparison (F-G). Genes with p < 0.001, calculated by empirical Bayes moderated t-test and false discovery rate adjustment, are included. Genes with M-value < 0 are up-regulated in differentiated cells, genes with M-value > 0 are up-regulated in neurospheres. M = log_2_(Cy5/Cy3); A = 1/2log_2_(Cy5*Cy3); p-value = unadjusted p-value; fdr adjusted p-value = false discovery rate adjusted p-value; B = B-value calculated by empirical Bayes moderated t-test. Higher B-values mean higher probability for differential expression. Click here for file
